# Femoral sciatic block: A safe alternative in von Recklinghausen’s disease

**DOI:** 10.4103/0019-5049.79885

**Published:** 2011

**Authors:** R Bhagyalakshmi, Rachel Cherian Koshy

**Affiliations:** Department of Anesthesia, Regional Cancer Center, Trivandrum, Kerala, India

Sir,

The neurofibromatoses are a group of autosomal dominant diseases, which are characterized by a tendency to form tumors of ectodermal and mesodermal tissues. They are of 2 main types NF1 and NF2.NF1 (von Recklinghausen’s neurofibromatosis) is the most common type. It is characterized by the presence of multiple neurofibromas and café au lait spots.[1,2]

A 56-year-old man, weighing 70 kg, with multiple neurofibromatosis presented at our center for excision of an ulceroproliferative lesion 15 × 10 cm in size on the dorsum of the foot followed by split skin grafting over the raw area. The skin over the lumbar area revealed large neurofibroma and scarring that resulted from previous excisions of neurofibroma. This precluded the use of lumbar neuraxial block. He also complained of dyspnea on exertion for the previous 2 months. CT evaluation revealed multiple soft tissue nodules suggestive of lung metastasis or neurofibromatosis. Hence a general anesthetic plan was not preferred, although the patient’s pulmonary function tests were normal. The patients’ blood investigation results were within normal limits. Physical examination on the day of surgery revealed pulse rate 80/min and blood pressure 130/80 mmHg. Chest was clear on auscultation.

**Figure 1:**
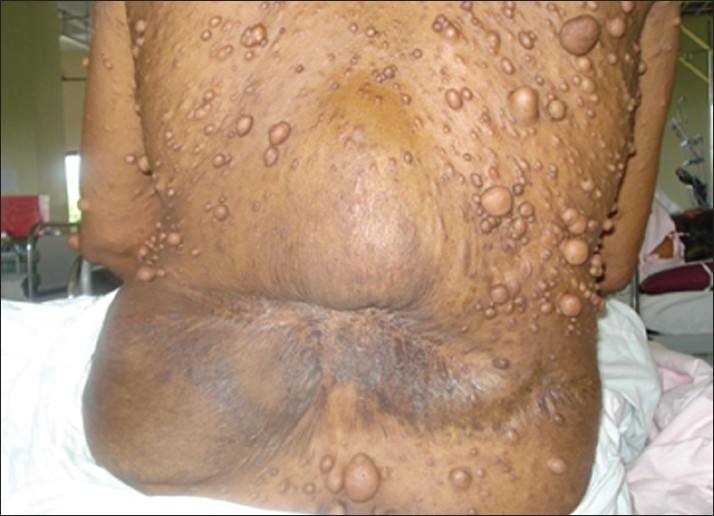
Multiple neurofibromas over lumbar region and scarring due to previous surgery

**Figure 2:**
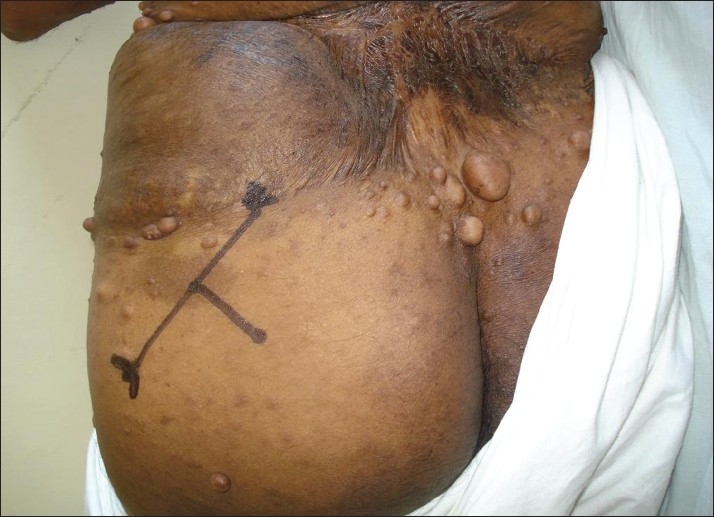
Landmarks for sciatic nerve block

**Figure 3:**
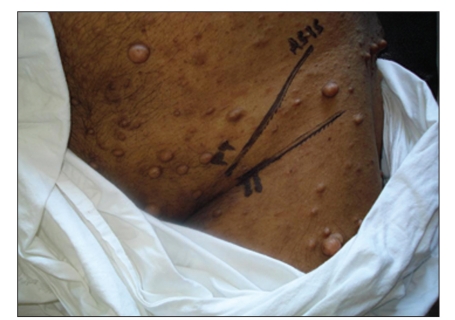
Landmarks for femoral nerve block

A combined sciatic and femoral block was performed guided by a nerve stimulator (Stimuplex^®^ Dig RC). The patient was reassured and premedicated with 2 mg midazolam and 100 mg fentanyl. After infiltrating local anesthetic, sciatic nerve block (classical posterior approach)[3] was achieved with 20 cc of 0.5% bupivacaine [Figures 1–3]

Femoral nerve block (identified by patellar twitch)[3] was given with 20 cc 2% lignocaine with adrenaline Onset of analgesia occurred in 15–20 min. Motor blockade on the foot was ascertained by the patient’s inability to move his toes. Supplemental oxygen was administered via a face mask. The surgery lasted for 2 h. The patient remained comfortable during the procedure.

The pain relief lasted for 4 h postoperatively. Postoperative pain was managed with intravenous administration of tramadol. The postoperative period was uneventful.

NF1 has an approximate incidence of 1 in 3000 births.[2,4] It is progressive, worsens with pregnancy[2,4] and during puberty and is more frequent among males.[2] Anesthetic management of patients with NF1 requires attention to all possible abnormalities and associated disturbances. Diffuse involvement of the tongue may lead to macroglossia. Lesions in the pharynx may complicate laryngoscopy and intubation or cause airway obstruction on induction. Pheochromocytoma is present in up to 1% of these patients accounting for secondary hypertension.[2,4] Gliomas, meningiomas, hydrocephalus, and spina bifida have been found in von-Recklinghausen’s patients.[5] This may be a relative contraindication to central neuraxial block. Vertebral deformities, such as scoliosis and spinal cord tumors, make spinal/extradural techniques difficult.[1] Severe spinal deformity may cause restrictive lung disease. Prolonged apnea has been reported with both depolarizing and nondepolarizing muscle relaxants. Current thought, however, is that these patients respond normally to neuromuscular blockers.[1]

Hence the choice of general or regional anesthesia is complex. Thus regional technique using a combined femoral and sciatic block is a safe and viable alternative for procedures on the leg and foot.
